# Impact of SARS-CoV-2 spike stability and RBD exposure on antigenicity and immunogenicity

**DOI:** 10.1038/s41598-024-56293-x

**Published:** 2024-03-08

**Authors:** Lucy Rutten, Maarten Swart, Annemart Koornneef, Pascale Bouchier, Sven Blokland, Ava Sadi, Jarek Juraszek, Aneesh Vijayan, Sonja Schmit-Tillemans, Johan Verspuij, Ying Choi, Chenandly E. Daal, Aditya Perkasa, Shessy Torres Morales, Sebenzile K. Myeni, Marjolein Kikkert, Jeroen Tolboom, Daniëlle van Manen, Harmjan Kuipers, Hanneke Schuitemaker, Roland Zahn, Johannes P. M. Langedijk

**Affiliations:** 1 Janssen Vaccines and Prevention B.V., Archimedesweg 4-6, Leiden, The Netherlands; 2 Molecular Virology Laboratory, Department of Medical Microbiology, Leiden University Medical Center, Leiden, The Netherlands; 3 Present Address: ForgeBio, Amsterdam, The Netherlands

**Keywords:** Protein vaccines, Immunology

## Abstract

The spike protein (S) of SARS-CoV-2 induces neutralizing antibodies and is the key component of current COVID-19 vaccines. The most efficacious COVID-19 vaccines are genetically-encoded spikes with a double proline substitution in the hinge region to stabilize S in the prefusion conformation (S-2P). A subunit vaccine can be a valuable addition to mRNA and viral vector-based vaccines but requires high stability of spike. In addition, further stabilization of the prefusion conformation of spike might improve immunogenicity. To test this, five spike proteins were designed and characterized, ranging from low to high stability. The immunogenicity of these proteins was assessed in mice, demonstrating that a spike (S-closed-2) with a high melting temperature, which still allowed ACE2 binding, induced the highest neutralization titers against homologous and heterologous strains (up to 16-fold higher than the least stabilized spike). In contrast, the most stable spike variant (S-locked), in which the receptor binding domains (RBDs) were locked in a closed conformation and thus not able to breathe, induced relatively low neutralizing antibody titers against heterologous strains. These data demonstrate that S protein stabilization with RBDs exposing highly conserved epitopes may be needed to increase the immunogenicity of spike proteins for future COVID-19 vaccines.

## Introduction

The trimeric spike glycoprotein (S) on the surface of SARS-CoV-2 is the primary target for neutralizing antibodies and the key component of most approved vaccines to combat COVID-19. Like other class I fusion proteins, S is intrinsically metastable and undergoes extensive conformational changes that are required to drive fusion and infect cells. Due to cleavage of S by furin into an S1 and an S2 subunit, and after binding of the receptor binding domain (RBD) to the angiotensin converting enzyme 2 receptor (ACE2), S transitions from an instable prefusion conformation to a stable postfusion conformation. The prefusion conformation is the main target for neutralizing antibodies and it has been shown for multiple fusion proteins to be the conformation that induces the highest neutralizing antibody titers. Hence this conformation is the primary target for vaccine development^1^.

Stabilization of S in the prefusion conformation has been achieved by proline substitutions of two consecutive residues (2P) in the hinge region of the S2 subunit between the central helix (CH) and heptad repeat 1 (HR1), which prevent the conformational change of spike into the postfusion state^2^. This 2P stabilization is used in full-length spikes in vaccines like Comirnaty (Pfizer-BioNTech), SpikeVax (Moderna), Jcovden (Janssen) and Nuvaxovid (Novavax), of which the first three are genetic vaccines and the fourth is purified protein^3,4^. Furin cleavage site mutations have been added to the spike in Jcovden and Nuvaxovid vaccines to prevent S1 from detaching from S2. In addition, a soluble purified S-2P protein containing a fibritin foldon trimerization domain has been tested in clinical trials by Sanofi (CoV-2 preS dTM^5^). Expression studies using S-2P indicated the design is metastable and additional stabilization strategies need to be applied to preserve the prefusion conformation^6–9^. Stabilization of the prefusion spike typically increases protein expression^2,6,7^ and improves the potency of eliciting neutralizing immune responses^4,8,10^.

The RBD of the SARS-CoV-2 spike protein is immunodominant. This domain is believed to be particularly important for generating neutralizing antibodies, as anti-RBD antibodies make up most of the neutralizing activity in human convalescent sera^11^. The RBD can adopt either a ‘closed’ state (also known as ‘down’) or an ‘open’ state (also known as ‘up’). In the open state, ACE2 can access the ACE2 receptor binding motif (RBM) and in the closed state it is shielded. Class 1 RBD-binding antibodies compete with ACE2 binding and can only bind to the RBD when the RBD is up, whereas class 2 and 3 RBD-binding antibodies bind to the RBD irrespective of whether it is up or down^12^. Antibodies that belong to class 4 RBD-binding antibodies bind to the inner face of the RBD, which is only exposed in the open state. These class 4 RBD-binding antibodies have the lowest neutralization potency but the highest neutralization breadth, due to higher conservation of the epitope^12^.

To investigate the effect of spike stabilization and RBD exposure on immunogenicity, we have developed recombinant SARS-CoV-2 spikes that were stabilized in either the closed or open conformation and measured induction of neutralizing antibodies in mice. The results show that an increase of the melting temperature of the spike increases induction of autologous neutralizing antibodies. However, for neutralization breadth it is important that the RBD is able to adopt an open conformation, which exposes the ACE2 binding site and the more conserved inner face of the RBD.

## Results

### Design of five differently stabilized spikes

To evaluate the impact of spike stability and RBD exposure on immunogenicity, we generated five spikes containing different sets of stabilizing substitutions. S-3P is a spike protein with a C-terminal foldon trimerization domain, a double proline substitution in the hinge loop (2P), substitutions in the furin cleavage site^2^ and an additional proline substitution at position 942 (A942P) that resulted in a strong increase in expression level compared with S-2P, without impacting melting temperature or the conformation of the trimer^6,7^. The potential impact of A942P on immunogenicity was evaluated by injecting mice intramuscularly with either S-2P or S-3P with AlPO_4_ as adjuvant. Spike-binding antibodies were determined 55 days later and showed no difference between S-2P and S-3P (Fig. S1). In addition, mice received S-3P dosed at three different dose levels (0.5, 5, and 50 µg) adjuvanted with AlPO_4_ or Al(OH)_3_. We observed that spike proteins adjuvanted with Al(OH)_3_ induced higher antibody titers than those adjuvanted with AlPO_4_ (Fig. S1). Therefore, we selected Al(OH)_3_ for further studies.

**Figure 1:**
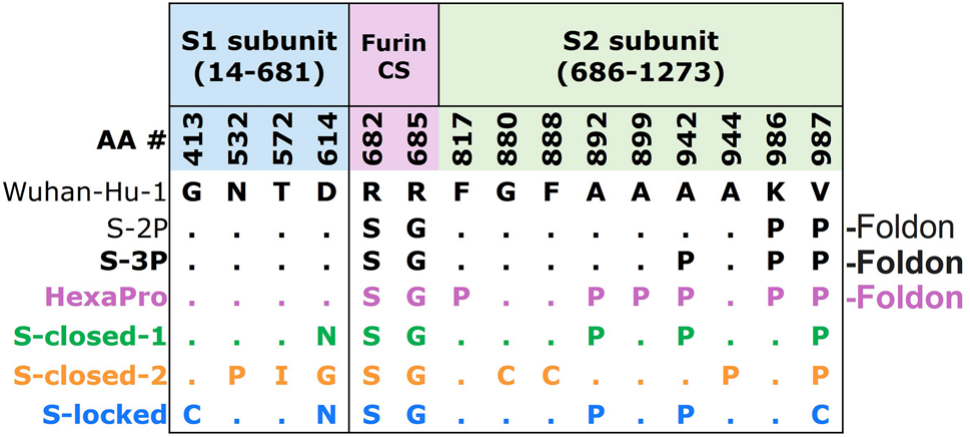
Design of five SARS-CoV-2 S proteins with different stabilizing substitutions. Table with substitutions applied to the different S proteins compared with the Wuhan-Hu-1 sequence. CS; cleavage site. The S-3P and HexaPro have, like the S-2P, foldon trimerization domains at the C-termini.

The S-3P protein was compared to spike versions with several additional stabilizing mutations that have been reported previously; HexaPro carrying a foldon domain^7^, S-closed-1^6^ and S-closed-2^6^, and S-locked, containing a disulfide that locks the RBD in the closed conformation (413C–987C)^8^ (Fig. 1 and Fig. S2). The latter three S proteins were designed without a heterologous trimerization domain in order to avoid potential off-target anti-foldon responses, which may increase upon repeated vaccination with foldon-containing proteins.

### Stabilized spike production and characterization

**Figure 2:**
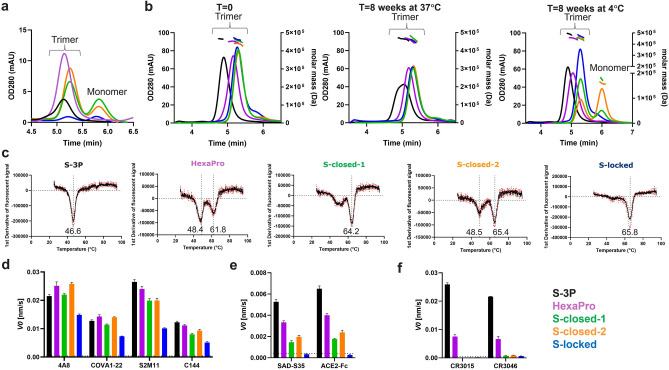
Expression and characterization of differently stabilized purified S proteins. (**A**) Analytical SEC on supernatants, reflecting trimer expression levels. (**B**) SEC profile of purified proteins at t = 0 (left panel), t = 8 weeks at 37 °C (middle panel) and t = 8 weeks at 4 °C (right panel). The molar mass traces determined by MALS signals are shown above the SEC curves. See Table S1 for theoretical and calculated MW and radiï. (**C**) First derivatives of fluorescent signals measured with differential scanning fluorimetry (DSF) with the black curve showing the average of a technical triplicate. The average of three values of calculated melting temperatures in °C are shown below the valleys in the curves. (**D**) Biolayer Interferometry (BLI) showing the initial slope *V*_*0*_ at the start of binding with neutralizing antibodies 4A8, COVA1-22 that bind to the NTD, and S2M11 and C144 that bind to the RBD irrespective of its position (up or down). (**E**) BLI with SAD-S35 and the ACE2 receptor that bind to the RBD when it is in the up position. (**F**) BLI with non-neutralizing antibodies CR3015 and CR3046 that bind epitopes in S2 that are shielded in the prefusion conformation. (**D**–**F**) Bars are plotted as average + SD (n = 3 technical replicates). The lower limit of detection (LLOD) is indicated with a horizontal dotted line. (**A**,**B**,**D**–**F**) Line and bar colors correspond to S protein legend in the lower right panel.

Trimer expression of the five S protein versions was evaluated in cell culture supernatant by analytical size exclusion chromatography (SEC) (Fig. 2A). HexaPro expressed approximately fourfold higher compared with S-3P, and as expected, both were trimeric since they were fused to the foldon trimerization domain. The two spike proteins S-closed-1 and S-closed-2 expressed primarily as trimers, with longer retention times in SEC than HexaPro and S-3P, due to the lack of the trimerization domain. Of these, S-closed-2 showed the highest trimer expression and the highest trimer:monomer ratio (Fig. 2A). The 413C–987C disulfide in S-locked significantly reduced overall expression levels.

Spike trimers were purified using lectin-affinity chromatography followed by SEC, and the quaternary structure was measured directly after purification and after 8 weeks of storage at 4 °C and 37 °C with SEC-MALS (Fig. 2B). Directly after purification all proteins were predominantly trimers with retention times that were similar to those observed in the cell culture supernatant, except for S-3P which had a shorter retention time, possibly indicating that the trimer opened up upon purification.

After 8 weeks storage at 4 °C, a small monomeric fraction appeared for S-closed-1 and a larger one for S-closed-2. In contrast, S-locked carrying interprotomeric disulfide 413C–987C did not show any monomers after 8 weeks storage at 4 °C. Upon prolonged storage at 4 °C, the retention time of all trimers remained unchanged, except for HexaPro. The HexaPro trimer shifted to a shorter retention time, suggesting that the trimer partially opens during storage at 4 °C. This phenomenon called ‘cold denaturation’, which causes the opening up of the foldon-linked spikes and the dissociation of the foldon-less spikes at 4 °C has been described before for SARS-CoV-2 spikes^9,13^. To better understand the dissociation as a result of cold denaturation, expression of HexaPro spike without foldon was tested using analytical SEC (Fig. S3A) and assessed after storage for 8 weeks at 4 °C and 37 °C (Fig. S3B). Foldon-less HexaPro almost completely dissociated into monomers after 8 weeks storage at 4 °C, demonstrating its sensitivity to cold denaturation. In addition, prolonged storage at 37 °C resulted in the appearance of a monomeric fraction.

In contrast, storage for 8 weeks at 37 °C of foldon-containing S3-P and HexaPro, and S-closed-1, S-closed-2, and S-locked immunogens did not lead to monomer formation. The S-3P trimers demonstrated a partial shift towards a longer retention time, which could be due to a partial closing of the trimers at 37 °C, in congruence with the observation that open trimers can close again upon incubation at 37 °C^9,13^. Freeze–thaw (FT) stability was measured with analytical SEC after snap freezing the spike proteins in liquid nitrogen, which demonstrated full trimer recovery for all spikes after one FT cycle, and slightly reduced recovery after five FT cycles (Fig. S4). The melting temperature (Tm_50_) of the different spike proteins was measured using differential scanning fluorimetry (DSF). Three of the five spike proteins showed two distinct melting events (Fig. 2C), one around 47 °C and another above 60 °C. S-3P and S-locked showed one melting event, with S-3P having the lowest melting temperature (Tm_50_ 46.6 °C) and S-locked having the highest Tm_50_ (65.8 °C) of all the spike proteins. The major melting temperature of HexaPro without foldon is 47.7 °C with a minor melting event at 63.3 °C (Fig. S3C).

In summary, the S-3P version with the foldon trimerization domain was produced likely as a closed trimer but appeared to convert into an open conformation upon purification, which could be partially reversed by incubation at 37 °C. The foldon-containing HexaPro trimer could be purified as a closed trimer, but still likely converted to a more open conformation upon prolonged storage at 4 °C. S-closed-1 and S-closed-2 dissociated into monomers over time at 4 °C whereas S-locked expressed poorly but retained a trimeric conformation after purification and prolonged storage at 4 °C and 37 °C.

### Antigenicity of differently stabilized spikes

The antigenicity of the purified spike trimers was measured with biolayer interferometry (BLI), using a panel of SARS-CoV-2 antibodies. The panel included neutralizing antibodies that recognize epitopes independent of the quaternary folding of the spike, i.e. 4A8 and COVA1-22, antibodies against the N-terminal domain (NTD) and S2M11 and C144 against the RBD. In order to measure the exposure of the RBD, binding was tested with antibody SAD-S35 and the ACE2 receptor which only bind the RBD when it is in the open (up) conformation (Fig. 2D,E and Fig. S5). To measure the exposure of cryptic epitopes on S2, binding was tested with non-neutralizing antibodies CR3015 and CR3046 that recognize epitopes in S2 that are shielded in the prefusion conformation and are exposed when the spike is in a more open conformation (Fig. 2F and Fig. S5). All proteins bound to the neutralizing antibodies 4A8, COVA1-22, S2M11 and C144. S-closed-1, S-closed-2 and S-locked showed little to no binding to the non-neutralizing antibodies. In contrast, HexaPro and S-3P respectively showed moderate and high binding to non-neutralizing CR3015 and CR3046. Removal of foldon from HexaPro reduced binding to these non-neutralizing antibodies (Fig. S3D). Possibly, HexaPro-foldon protein contained a fraction of suboptimally folded (‘open’) trimers that could not be separated during purification, in contrast to foldon-less HexaPro which clearly separated into trimeric and monomeric fractions (Fig. S3A).

Binding to ACE2 and SAD-S35 was abolished for S-locked, in agreement with the reported down conformation of the RBD caused by introduction of disulfide 413C–987C^8^. Binding of SAD-S35 and ACE2 was also reduced for HexaPro, S-closed-1, and S-closed-2 compared to S-3P, indicating a more open conformation of the S3-P protein.

In summary, BLI data confirmed that S-closed-1 and S-closed-2 proteins are in a closed conformation, as shown by the lack of non-neutralizing antibody binding, but have breathing RBDs able to move up and down, as shown by ACE2 and SAD-S35 binding. In contrast, S-locked is in a locked conformation, as shown by absence of non-neutralizing antibody binding and abrogated ACE2 and SAD-S35 binding.

### Immunogenicity of SARS-CoV-2 spike proteins with different stabilization

**Figure 3:**
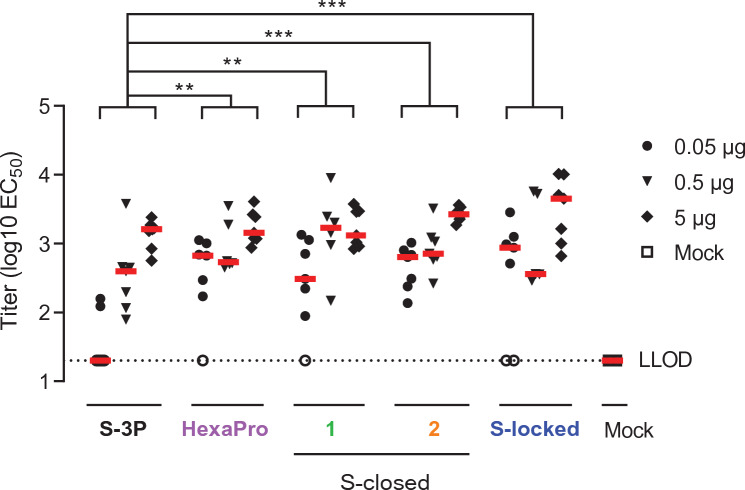
SARS-CoV-2 spike-binding antibodies in mice immunized with recombinant spike constructs using a Wuhan-Hu-1 RBD ELISA. Mice were immunized at day 0 and 28 with 0.05 to 5 µg S-3P, HexaPro, S-closed-1, -2, or S-locked protein adjuvanted with 100 µg Al(OH)_3_ or PBS as a mock control. Spike Wuhan-Hu-1 RBD-binding antibody titers were measured by ELISA at day 56. Red horizontal lines indicate the median response per group and the dotted line indicates the lower limit of detection (LLOD). Open symbols indicate the response is at or below the LLOD. Data were analyzed by a pairwise comparison across dose with a z-test from Tobit ANOVA with a tenfold Bonferroni correction. Significant differences between the protein-vaccinated groups are shown. **p < 0.01; ***p < 0.001.

**Figure 4:**
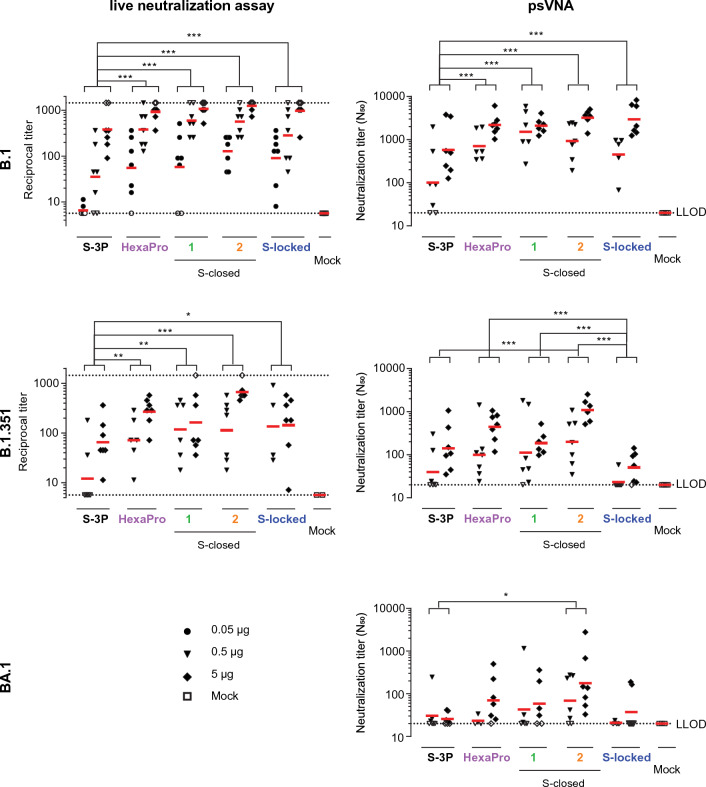
Neutralization titers in mouse sera against SARS-CoV-2 B.1, B.1.351 and BA.1. Mice were immunized at day 0 and 28 with 0.05 to 5 µg S-3P, HexaPro, S-closed-1, -2, or S-locked protein adjuvanted with 100 µg Al(OH)_3_ or PBS as a mock control. Neutralizing antibody titers were measured with a live virus VNA or a pseudotyped virus (psVNA) against SARS-CoV-2 B.1, B.1.351 and BA.1. Red horizontal bars indicate the median response per group and the dotted lines indicate the lower and upper limits of detection (L/ULOD). Open symbols indicate response is at L/ULOD. Data were analyzed by a pairwise comparison across dose with a z-test from Tobit ANOVA or a Cochran–Mantel–Haenszel test with a tenfold Bonferroni correction. Significant differences between the protein-vaccinated groups are shown at the top of the graphs. *p < 0.1, **p < 0.01; ***p < 0.001.

To evaluate the immunogenicity of the stabilized spike proteins, mice received a two-dose regimen at day 0 and 28 with 0.05, 0.5 or 5 µg of each of the spike proteins adjuvanted with aluminum hydroxide [Al(OH)_3_]. The spike proteins were snap frozen in liquid nitrogen, stored at − 80 °C, and administered immediately after thawing to preserve the trimeric structure. SARS-CoV-2 RBD-binding antibodies and neutralizing antibodies were measured 4 weeks after the second dose. The more stabilized spikes HexaPro, S-closed-1, S-closed-2 and S-locked elicited higher SARS-CoV-2 RBD antibody-binding titers than S-3P as measured by ELISA, especially at the lowest antigen dose tested (Fig. 3). In addition, animals immunized with these spikes had up to 16-fold higher neutralizing antibody titers compared with animals immunized with the open S-3P, as measured using both a live virus neutralization assay and a pseudovirus VNA (psVNA) based on the B.1 strain (Fig. 4). The more stabilized spike versions, in particular S-closed-2, also induced higher neutralizing antibody titers against the heterologous B.1.135 strain compared with S-3P as measured by live virus neutralization assay. While S-locked also induced higher B.1.351 neutralizing antibodies than S-3P in a live virus neutralization assay, B.1.351 neutralization was not improved compared with S-3P in a B.1.351 psVNA assay. S-closed-2 showed significantly higher neutralization compared with S-3P against Omicron BA.1 (Fig. 4), although overall at lower levels than observed for B.1 and B.1.351.

## Discussion

The most efficacious approved COVID-19 vaccines to date contain a double proline substitution in the hinge region, which stabilizes full-length spikes in the prefusion conformation, and this has been shown to improve the induction of neutralizing antibodies^10,14^. With the aim to enhance immunogenicity and induce a broad neutralization response, we tested the impact of additional spike stabilizing mutations and RBD exposure in soluble spike subunit antigens. To this end, we designed and characterized five versions of full SARS-CoV-2 spike protein ectodomains with different sets of stabilizing substitutions, resulting in spikes ranging from low stability with high dynamic behavior (S-3P) to high stability with limited dynamic behavior and limited RBD exposure (S-locked). Although some spikes dissociated into monomers after storage at 4 °C, all spikes fully kept their trimeric structure after freeze-thawing and prolonged storage at 37 °C. Since spikes were used for immunization directly after thawing, the instability at 4 °C is not expected to have an impact on immunogenicity and thus not relevant for the comparison of spikes in mice.

We demonstrated that further stabilized spike proteins elicited higher RBD-binding antibody titers in mice than S-3P, which was chosen as a substitute for S-2P because of its much higher expression levels, without impacting immunogenicity. Importantly, the more stabilized closed spikes induced significantly higher titers of neutralizing antibodies against the B.1 strain, which is closely related to the strain from which the spike immunogens were derived, as measured by both a live virus neutralization assay and psVNA assays, compared the open S-3P immunogen.

Interestingly, the S-locked variant, with the highest melting temperature, showed the least improved induction of neutralization compared with the open S-3P against the heterologous B.1.351 (Beta) strain in live virus neutralization assay, and no improvement in psVNA. Likewise, S-locked induced low neutralizing titers against heterologous strain BA.1 (Omicron) in psVNA. We hypothesize that the S-locked predominantly induces E484-dependent class 2 RBD-binding antibodies against the closed RBD^12^. Therefore, the induction of neutralizing antibodies may have been hampered by the E484K mutation which is present in B.1.351^15,16^ and by escape mutations in BA.1 in the epitope of class 2 RBD-binding antibodies^17,18^. Conversely, stabilized S proteins that still allow movement of the RBD and thus expose the ACE2 binding site and the inner face of the RBD induce high titers of neutralizing antibodies that likely bind to conserved sites in the RBD which are occluded in the S-locked variant. S-closed-2 elicited the highest neutralization titers against heterologous strains B.1.351 and BA.1 in psVNA.

We postulate that apart from RBD exposure, the melting temperature of the spike protein is an important contributing factor for optimal induction of broad neutralizing antibodies. S-closed-2 showed the highest melting temperature of the ‘breathing’ stabilized spike variants, which may account for a longer half-life in the host and may also contribute to improved immunogenicity. Conversely, S-3P and to a lesser extent HexaPro demonstrated lower stability, indicated by lower melting temperatures and lower retention time, indicating a more open structure after purification and the exposure of epitopes for non-neutralizing antibodies. The lower stability of S-3P and relatively poor immunogenicity and neutralization response support the notion that less stable proteins may be more quickly degraded in vivo than more stable spikes.

Although the more stabilized spike proteins were stable after freeze-thawing and during prolonged storage at 37 °C, cold denaturation remains an issue to be addressed if it would be desired to store the vaccine at 4 °C. The occurrence of monomers of S-closed-1 and S-closed-2 upon prolonged storage at 4 °C might be mitigated by improving buffer formulation or by increasing protein stability even further. For instance, the introduction of an inter-protomeric disulfide bond in the S2 domain has been positively evaluated in the VFLIP S design^19^. Addition of a heterologous trimerization domain is not expected to solve the 4 °C stability issue, as the HexaPro variant carrying a foldon trimerization domain still showed a retention shift in SEC upon prolonged storage at 4 °C, suggesting destabilization and opening-up of the trimer conformation^13^. Moreover, the use of a heterologous foldon domain could prove disadvantageous after repeated immunization as an anti-foldon immune response might increase and could potentially hamper spike neutralization titers, especially in a human population immunized with e.g. foldon-stabilized prefusion proteins against other targets like the previously approved vaccines against respiratory syncytial virus^20–22^.

In summary, this study describes a strategy towards the development of a SARS-CoV-2 subunit vaccine candidate with desirable attributes. We designed and evaluated stabilized trimeric spike immunogens without a heterologous trimerization domain that remained stable under physiologically relevant circumstances. Finally, our data demonstrate that for induction of broad neutralization, antigen designs need movement of the RBD in order to expose conserved epitopes.

## Materials and methods

### Protein expression and purification

Plasmids encoding SARS-CoV-2 S protein were synthesized and codon-optimized at GenScript (Piscataway, NJ 08854). All S proteins contain amino acids 14-1208 of the Wuhan-Hu-1 SARS-CoV-2 spike (GenBank accession no. MN908947) and carry furin cleavage site mutations R682S and R685G. Plasmids were transiently expressed in Expi293F cells using ExpiFectamine (Life Technologies) according to the manufacturer’s instructions and cultured for three to five days at 37 °C and 10% CO_2_. Cell-free sterile-filtered cell culture supernatants were either directly applied to an analytical SEC column (see below) or subjected to a two-step purification protocol. Trimeric spike protein was purified by applying cleared culture supernatant on a *Galanthus nivalis* (GN)-lectin column (Vectorlabs, AL-1243) with 40 mM tris, 500 mM NaCl pH 7.4 as a running buffer. Elution was performed with the same buffer with additional 1 M mannopyranoside with a final pH of 7.4. Eluted protein was concentrated and subsequently loaded on a Superdex200 Increase column (GE Healthcare) (or HiLoad Superdex 200 16/600 column) with 20 mM tris, 150 mM NaCl pH 7.4 as running buffer. Sucrose was added to a final concentration of 5% before snap freezing in liquid nitrogen, in order to store the proteins at − 80 °C prior to immunization studies.

### Antibodies and reagents

SAD-S35 was purchased at Acro Biosystems. ACE2-Fc was made according to Liu et al.^23^. For 4A8, S2M11, C144, CR3015 and CR2046 the heavy and light chains were cloned into a single IgG1 expression vector to express a fully human IgG1 antibody. The antibodies were made by transfecting the IgG1 expression construct using the ExpiFectamine™ 293 Transfection Kit (ThermoFisher) in Expi293F (ThermoFisher) cells according to the manufacturer’s specifications. They were purified from serum-free culture supernatants using mAb Select SuRe resin (GE Healthcare) followed by rapid desalting using a HiPrep 26/10 Desalting column (GE Healthcare). The final formulation buffer was 20 mM NaAc, 75 mM NaCl, and 5% sucrose pH 5.5. COVA1-22 was a kind gift by Marit van Gils^24^.

### Analytical SEC

An ultra-high-performance liquid chromatography system (Vanquish, Thermo Scientific) and µDAWN TREOS instrument (Wyatt) coupled to an Optilab µT-rEX Refractive Index Detector (Wyatt), in combination with an in-line Nanostar DLS reader (Wyatt) were used to perform the analytical SEC experiment. The cleared crude cell culture supernatants were applied to an SRT-10C SEC-500 15 cm column (Sepax Cat. #235500-244615) with the corresponding guard column (Sepax) equilibrated in running buffer (150 mM sodium phosphate, 50 mM NaCl, pH 7.0) at 0.35 mL/min. When analyzing supernatant samples, µMALS detectors were offline and analytical SEC data were analyzed using the Chromeleon 7.2.8.0 software package. The signal of supernatants of non-transfected cells was subtracted from the signal of supernatants of S transfected cells. When purified proteins were analyzed using SEC-MALS, µMALS detectors were inline and data were analyzed using the Astra 7.3 software package.

### Biolayer interferometry (BLI)

Antibodies were immobilized at a concentration of 10 µg/mL on anti-hIgG (AHC) sensors (Sartorius, cat. #18-5060) in 1× kinetics buffer (Sartorius, cat. #18-1105) in 384-well black tilted-bottom polypropylene microplates (Sartorius cat. #18-5076). The experiment was performed in triplicate with an Octet HTX instrument (Sartorius) at 30 °C with a shaking speed of 1000 rpm. Sensor hydration was 600 s, immobilization of antibodies 600 s, followed by washing for 300 s, and then binding the S proteins at 10 μg/mL for 300 s and dissociation for 300 s. The data analysis was performed using FortéBio Data Analysis 12.0 software (Sartorius).

### Differential scanning fluorometry (DSF)

The fluorescent emission of Sypro Orange Dye (Thermo Fisher Scientific) added to S protein in solution was monitored. The measurement was performed with a starting temperature of 25 °C and a final temperature of 95 °C (54 °C increase per hour). Melting curves were measured using a ViiA7 real-time PCR machine (Applied Biosystems) and melting temperature (Tm_50_) values were derived from the negative first derivative as described previously^25^. The melting temperature corresponds to the lowest point in the curve.

### Mouse immunization

Animal experiments were approved by the Central Authority for Scientific Procedures on Animals (Centrale Commissie Dierproeven), the institutional animal welfare body, and conducted in accordance with the European guidelines (EU directive on animal testing 2010/63/EU and ETS 123) and local Dutch legislation on animal experiments and reported in accordance with the ARRIVE guidelines. Female BALB/c mice aged 8–12 weeks at the start of the study were purchased from Charles River Laboratories (Germany). Mice were vaccinated intramuscularly with 100 μL (50 μL per hind-leg) vaccine under general anesthesia with isoflurane. At the end of the study the animals were exsanguinated by cardiac puncture under isoflurane anesthesia and sacrificed by cervical dislocation.

### RBD ELISA

ELISA plates were coated with 2 μg/mL SARS-CoV-2 spike RBD protein from lineage B (Wuhan-Hu-1) in PBS O/N at 4 °C. The used RBD protein contains spike amino acids 319–543 and includes the tissue plasminogen activator (tPA) signal peptide and a C-tag (sequence EPEA) at its C-terminus. Then, the plates were washed with 3 × 150 μL PBS with 0.05% Tween-20 (PBST) and blocked with 1% casein in PBS for 1 h at room temperature (RT). Next, plates were washed again with 3 × 150 μL PBST. Subsequently, serum samples were added with a starting dilution of 1:20 and serially diluted threefold. Following an incubation for 1 h at RT, plates were washed with 3 × 150 μL PBST and goat-anti-mouse IgG HRP (1/20,000) was added for 1 h at RT. Finally, the plates were washed 3× with 150 μL PBST and then 20 μL detection substrate (Enhanced chemiluminescent; ECL) was added and incubated for 10 min. Luminescence was read on a BioTek Synergy Neo plate reader. Titers are reported as log10 of the half maximal concentration (EC50), the log10 dilution at which half maximal binding is reached. The LLOD was set at the log10 of the lowest dilution and the ULOD at the log10 of the highest dilution tested.

### Spike ELISA

ELISA plates were coated with 1 μg/mL SARS-CoV-2 spike from lineage B (Wuhan-Hu-1) in PBS O/N at 4 °C. Then, the plates were washed with 3 × 150 μL PBS with 0.05% Tween-20 (PBST) and blocked with 1% casein in PBS for 1 h at RT. Next, plates were washed again with 3 × 150 μL PBST. Subsequently, serum samples were added with a starting dilution of 1:100 and serially diluted threefold. Following an incubation for 1 h at RT, plates were washed with 3 × 150 μL PBST and goat-anti-mouse IgG HRP (1/20,000) was added for 1 h at RT. Finally, the plates were washed 3× with 150 μL PBST and then 20 μL detection substrate (Enhanced chemiluminescent; ECL) was added and incubated for 10 min. Luminescence was read on a BioTek Synergy Neo plate reader. Titers are reported as log10 endpoint titers.

### Recombinant lentivirus-based pseudotyped virus neutralization assay (psVNA)

Neutralizing antibody titers were measured against several SARS-CoV-2 spike versions by a pseudotyped virus neutralization assay (psVNA). Human Immunodeficiency Virus (HIV)-based lentiviruses, pseudotyped with SARS-CoV-2 spike protein (based on WuhanHu1; GenBank accession number MN908947) were generated as described previously^26^. Substitutions and deletions in the spike protein open reading frame for the strains B.1, B.1.351 (Beta) and BA.1 (Omicron) were introduced using standard molecular biology techniques and confirmed by sequencing.

Assays were performed on HEK293T target cells stably expressing the human angiotensin-converting enzyme 2 (ACE2) and human transmembrane serine protease 2 (TMPRSS2) genes (VectorBuilder). The cells were seeded in white half-area 96-well tissue culture plates (Perkin Elmer) at a density of 1.5 × 10^4^ cells/well. Two-fold serial dilutions were prepared from heat-inactivated serum samples in phenol red-free Dulbecco's Modified Eagle Medium (DMEM) supplemented with 1% FBS and 1% penicillin/streptomycin. The serially diluted serum samples were incubated at RT with an equal volume of pseudoviral particles with titers of approximately 1 × 10^5^ Relative Luminescence Units (RLU) luciferase activity. After one hour incubation, the serum-particle mixture was inoculated onto HEK293T.ACE2.TMPRSS2 cells. Luciferase activity was measured 40 h after transduction by adding an equal volume of NeoLite substrate (Perkin Elmer) to the wells according to the manufacturer’s protocol, followed by read out of RLU on the EnSight Multimode Plate Reader (Perkin Elmer). SARS-CoV-2 neutralizing titers were calculated using a four-parameter curve fit as the sample dilution at which a 50% reduction (N50) of luciferase readout was observed compared with luciferase readout in the absence of serum (High Control). The starting serum sample dilution of 20 was fixed as the lower limit of detection (LLOD).

### Live virus neutralization assay

Live virus neutralization titers were determined at the Leiden University Medical Center (LUMC, the Netherlands). Isolate SARS-CoV-2/human/NLD/Leiden-0008/2020 (lineage B.1, GenBank accession number: MT705206.1) was propagated and titrated in Vero E6 cells. hCoV-19/Belgium/rega-1920/2021; EPI_ISL_896474, 2021-01-11 (lineage B.1.351) was a generous gift of Prof. Dr. Piet Maes, Laboratory of Clinical and Epidemiological Virology, dept. of Microbiology, Immunology and Transplantation, Rega Institute for Medical Research, Leuven, Belgiu and was propagated on Calu-3 cells. The 50% tissue culture infectious dose (TCID50) endpoint dilution method and the TCID50 were calculated by the Spearman-Kärber algorithm as previously described by Hierholzer and Killington^27^. Vero-E6 cells were seeded at 12,000 cells/well in 96-well tissue culture plates one day prior to infection. Heat-inactivated (60 min at 56 °C) serum samples were analyzed in duplicate. The panel of sera were two-fold serially diluted in duplicate, with an initial dilution of 1:10 and a final dilution of 1:1280 in 60 μL Eagle's Minimum Essential Medium (EMEM) supplemented with penicillin, streptomycin, 2 mM l-glutamine and 2% fetal calf serum. Diluted sera were mixed with equal volumes of 120 TCID50/60 μL Leiden-0008 (lineage B.1) virus and incubated for 1 h at 37 °C. The virus-serum mixtures were then added onto Vero E6 cell monolayers and incubated at 37 °C in a humidified atmosphere with 5% CO_2_. Cells either unexposed to the virus or mixed with 120 TCID50/60 μL SARS-CoV-2 were used as negative (uninfected) and positive (infected) controls, respectively. At three days post infection, cells were fixed and inactivated with 40 μL 37% formaldehyde/PBS solution/well overnight at 4 °C. The fixative was removed from cells and the clusters were stained with 50 μL/well crystal violet solution, incubated for 10 min and rinsed with water. Dried plates were evaluated for viral cytopathic effect. Neutralization titers were calculated by dividing the number of positive wells with complete inhibition of the virus-induced cytopathogenic effect, by the number of replicates, and adding 2.5 to stabilize the calculated ratio. The neutralizing antibody titer was defined as the log2 reciprocal of this value. A SARS-CoV-2 back-titration was included with each assay run to confirm that the dose of the used inoculum was within the acceptable range of 30 to 300 TCID50.

### Supplementary Information

Supplementary Information.

## Data Availability

The analyzed data for this study are included in this published article. Raw data supporting the findings can be made available by the corresponding author upon a reasonable request.
