# Supplementary material for: Impact of SARS-CoV-2 spike stability and RBD exposure on antigenicity and immunogenicity
Source: Sci Rep. 2024 Mar 8;14:5735. doi: 10.1038/s41598-024-56293-x (PMC10923862; doi:10.1038/s41598-024-56293-x)
Supplement: Supplementary file 1 — Supplementary Information. [file 41598_2024_56293_MOESM1_ESM.pdf]

# Supplementary Information

## Impact of SARS-CoV-2 spike stability and RBD exposure on antigenicity and immunogenicity

Lucy Rutten[1], Maarten Swart[1], Annemart Koornneef[1], Pascale Bouchier[1], Sven Blokland[1], Ava Sadi[1], Jarek Juraszek[1], Aneesh Vijayan[1], Sonja Schmit-Tillemans[1], Johan Verspuij[1], Ying Choi[1], Chenandly E. Daal[1], Aditya Perkasa[1], Shessy Torres Morales[2], Sebenzile K. Myeni[2], Marjolein Kikkert[2], Jeroen Tolboom[1], Daniëlle van Manen[1], Harmjan Kuipers[1], Hanneke Schuitemaker[1], Roland Zahn[1] and Johannes P.M. Langedijk[1,3*]

[1]Janssen Vaccines & Prevention B.V., Archimedesweg 4-6, Leiden, The Netherlands.
[2]Molecular Virology Laboratory, Department of Medical Microbiology, Leiden University Medical Center, Leiden, The Netherlands.
[3]Current affiliation: ForgeBio, Amsterdam, The Netherlands
[*]Corresponding author. E-mail: hlangedijk@forge-bio.com

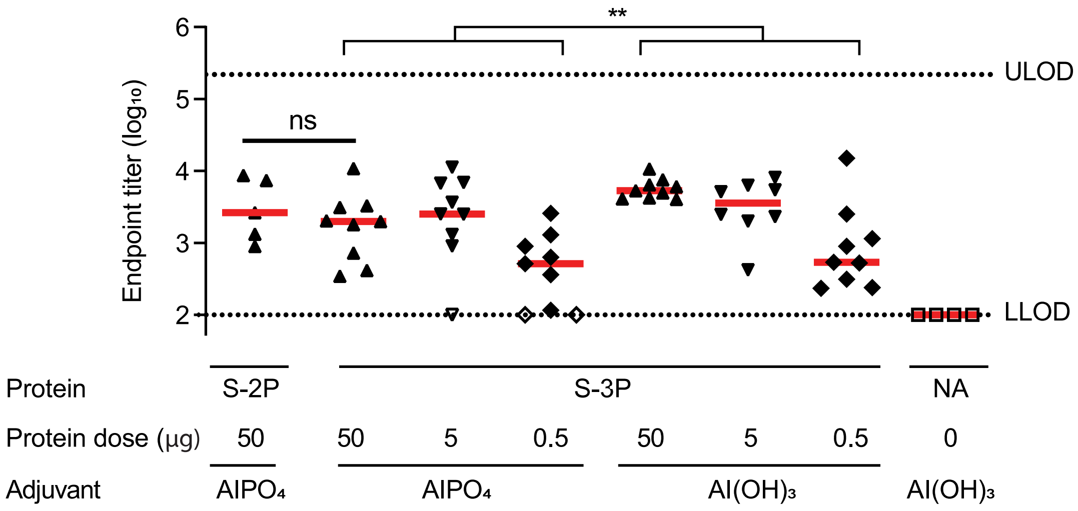


**Supplementary Figure S1. Immunogenicity of S-3P with $Al(OH)_3$ and $Al(PO)_4$.** Mice were immunized at day 0 with 0.5, 5 or 50 µg S-3P adjuvanted with 100 µg $Al(OH)_3$ or $AlPO_4$ or 50 µg S-2P with 100 µg $AlPO_4$. Spike-binding antibody titers were evaluated with ELISA at day 55. Red horizontal bars indicate the median response per group and the dotted lines indicates the upper and lower limits of detection (U/LLOD). Open symbols indicate response is at U/LLOD. 50 µg S-2P and S-3P adjuvanted with $AlPO_4$ were compared by a t-test. Differences between $Al(OH)_3$ and $AlPO_4$ were analyzed by a pairwise comparison across doses with a z-test from Tobit ANOVA. ns, not significant; **, $p<0.01$.

```
S-3P         MFVFLVLLPLVSSQCVNLTTRTQLPPAYTNSFTRGVYYPDKVFRSSVLHSTQDLFLPFFSNVTWFHAIHVSGTNGTKRFDNPVLPFNDGVYFASTEKSNI 100
HexaPro      .................................................................................................... 100
S-closed-1   .................................................................................................... 100
S-closed-2   .................................................................................................... 100
S-locked     .................................................................................................... 100

S-3P         IRGWIFGTTLDSKTQSLLIVNNATNVVIKVCEFQFCNDPFLGVYYHKNNKSWMESEFRVYSSANNCTFEYVSQPFLMDLEGKQGNFKNLREFVFKNIDGY 200
HexaPro      .................................................................................................... 200
S-closed-1   .................................................................................................... 200
S-closed-2   .................................................................................................... 200
S-locked     .................................................................................................... 200

S-3P         FKIYSKHTPINLVRDLPQGFSALEPLVDLPIGINITRFQTLLALHRSYLTPGDSSSGWTAGAAAYYVGYLQPRTFLLKYNENGTITDAVDCALDPLSETK 300
HexaPro      .................................................................................................... 300
S-closed-1   .................................................................................................... 300
S-closed-2   .................................................................................................... 300
S-locked     .................................................................................................... 300

S-3P         CTLKSFTVEKGIYQTSNFRVQPTESIVRFPNITNLCPFGEVFNATRFASVYAWNRKRISNCVADYSVLYNSASFSTFKCYGVSPTKLNDLCFTNVYADSF 400
HexaPro      .................................................................................................... 400
S-closed-1   .................................................................................................... 400
S-closed-2   .................................................................................................... 400
S-locked     .................................................................................................... 400

S-3P         VIRGDEVRQIAPGQTGKIADYNYKLPDDFTGCVIAWNSNNLDSKVGGNYNYLYRLFRKSNLKPFERDISTEIYQAGSTPCNGVEGFNCYFPLQSYGFQPT 500
HexaPro      .................................................................................................... 500
S-closed-1   .................................................................................................... 500
S-closed-2   .................................................................................................... 500
S-locked     ...........C........................................................................................ 500

S-3P         NGVGYQPYRVVVLSFELLHAPATVCGPKKSTNLVKNKCVNFNFNGLTGTGVLTESNKKFLPFQQFGRDIADTTDAVRDPQTLEILDITPCSFGGVSVITP 600
HexaPro      .................................................................................................... 600
S-closed-1   .................................................................................................... 600
S-closed-2   ..............................P........................................I............................ 600
S-locked     .................................................................................................... 600

S-3P         GTNTSNQVAVLYQDVNCTEVPVAIHADQLTPTWRVYSTGSNVFQTRAGCLIGAEHVNNSYECDIPIGAGICASYQTQTNSPSRAGSVASQSIIAYTMSLG 700
HexaPro      .................................................................................................... 700
S-closed-1   .............N...................................................................................... 700
S-closed-2   .............G...................................................................................... 700
S-locked     .............N...................................................................................... 700

S-3P         AENSVAYSNNSIAIPTNFTISVTTEILPVSMTKTSVDCTMYICGDSTECSNLLLQYGSFCTQLNRALTGIAVEQDKNTQEVFAQVKQIYKTPPIKDFGGF 800
HexaPro      .................................................................................................... 800
S-closed-1   .................................................................................................... 800
S-closed-2   .................................................................................................... 800
S-locked     .................................................................................................... 800

S-3P         NFSQILPDPSKPSKRSFIEDLLFNKVTLADAGFIKQYGDCLGDIAARDLICAQKFNGLTVLPPLLTDEMIAQYTSALLAGTITSGWTFGAGAALQIPFAM 900
HexaPro      ................P..........................................................................P......P. 900
S-closed-1   ...........................................................................................P........ 900
S-closed-2   ...............................................................................C.......C............ 900
S-locked     ...........................................................................................P........ 900

S-3P         QMAYRFNGIGVTQNVLYENQKLIANQFNSAIGKIQDSLSSTPSALGKLQDVVNQNAQALNTLVKQLSSNFGAISSVLNDILSRLDPPEAEVQIDRLITGR 1000
HexaPro      .................................................................................................... 1000
S-closed-1   .....................................................................................K.............. 1000
S-closed-2   .........................................A.P.........................................K.............. 1000
S-locked     .....................................................................................KC............. 1000

S-3P         LQSLQTYVTQQLIRAAEIRASANLAATKMSECVLGQSKRVDFCGKGYHLMSFPQSAPHGVVFLHVTYVPAQEKNFTTAPAICHDGKAHFPREGVFVSNGT 1100
HexaPro      .................................................................................................... 1100
S-closed-1   .................................................................................................... 1100
S-closed-2   .................................................................................................... 1100
S-locked     ....................................................................................................

S-3P         HWFVTQRNFYEPQIITTDNTFVSGNCDVVIGIVNNTVYDPLQPELDSFKEELDKYFKNHTSPDVDLGDISGINASVVNIQKEIDRLNEVAKNLNESLIDL 1200
HexaPro      .................................................................................................... 1200
S-closed-1   .................................................................................................... 1200
S-closed-2   .................................................................................................... 1200
S-locked     .................................................................................................... 1200

S-3P         QELGKYEQGSGYIPEAPRDGQAYVRKDGEWVLLSTFLLPATGGSEPEA 1248
HexaPro      ................................................ 1248
S-closed-1   ........---------------------------------------- 1208
S-closed-2   ........---------------------------------------- 1208
S-locked     ........---------------------------------------- 1208
```

**Supplementary Figure S2**. **Protein sequence alignment of five stabilized spikes.** The signal peptide is indicated with a green background. The foldon trimerization domain followed by a sortase A tag and C-tag is indicated with a cyan background. All substitutions compared to the ectodomain sequence of the Wuhan-Hu-1 spike are indicated with a yellow background. A dot indicates that the amino acid is the same as in the first sequence and a dash indicates that the residue is missing compared to the first sequence.

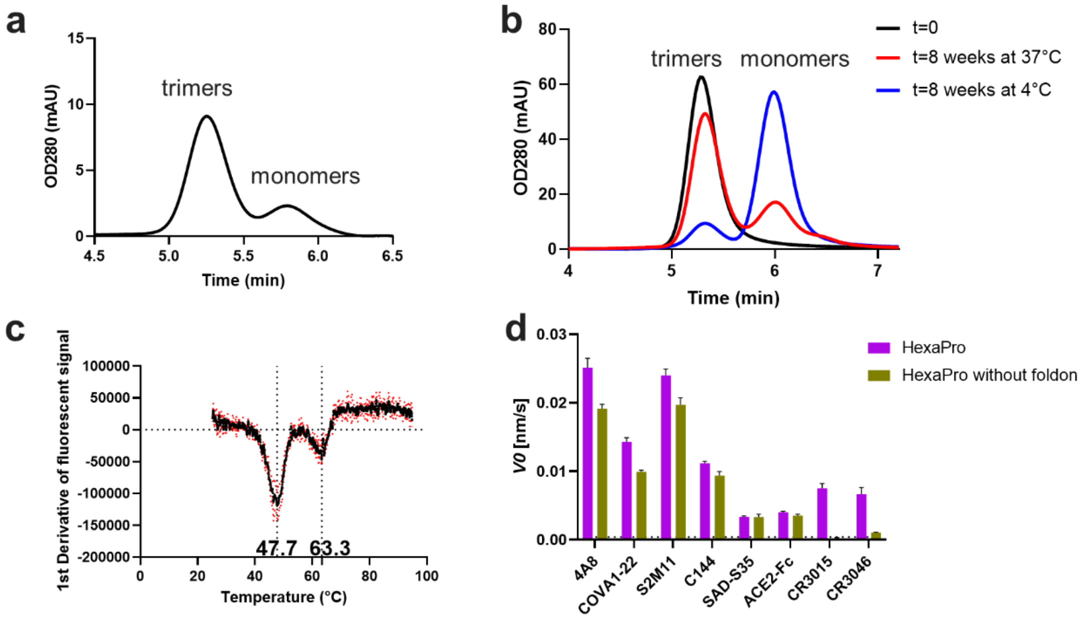


**Supplementary Figure S3. Expression and characterization of HexaPro without a foldon trimerization domain.** (**a**) Analytical SEC on supernatant, reflecting trimer expression levels. (**b**) SEC pattern of purified protein at t=0, t=8 weeks at 37°C, and t=8 weeks at 4°C. (**c**) First derivatives of fluorescent signals measured with differential scanning fluorimetry (DSF) with the black curve showing the average of a technical triplicate. The average of three values of calculated melting temperatures in °C are shown below the valleys in the curves. (**d**) Biolayer Interferometry (BLI) showing the initial slope V0 at the start of binding with neutralizing antibodies 4A8 and COVA1-22 that bind to the NTD, and S2M11 and C144 that bind to the RBD irrespective of its position (up or down), and SAD-S35 and the ACE2 receptor that bind to the RBD when it is in the up position, and non-neutralizing antibodies CR3015 and CR3046 that bind epitopes in S2 that are shielded in the prefusion conformation. The lower limit of detection (LLOD) is indicated with a horizontal dotted line. Bars are plotted as average +SD (n=3 technical replicates).

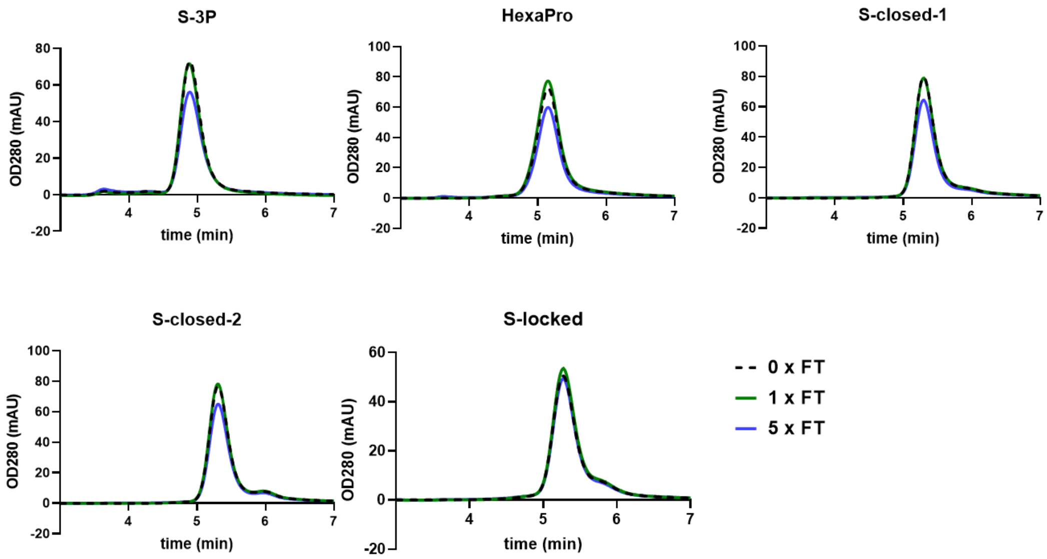


**Supplementary Figure S4. SEC analysis before and after freeze-thawing.** Flash freezing in liquid nitrogen and thawing (freeze-thawing; FT) was performed one and five times.

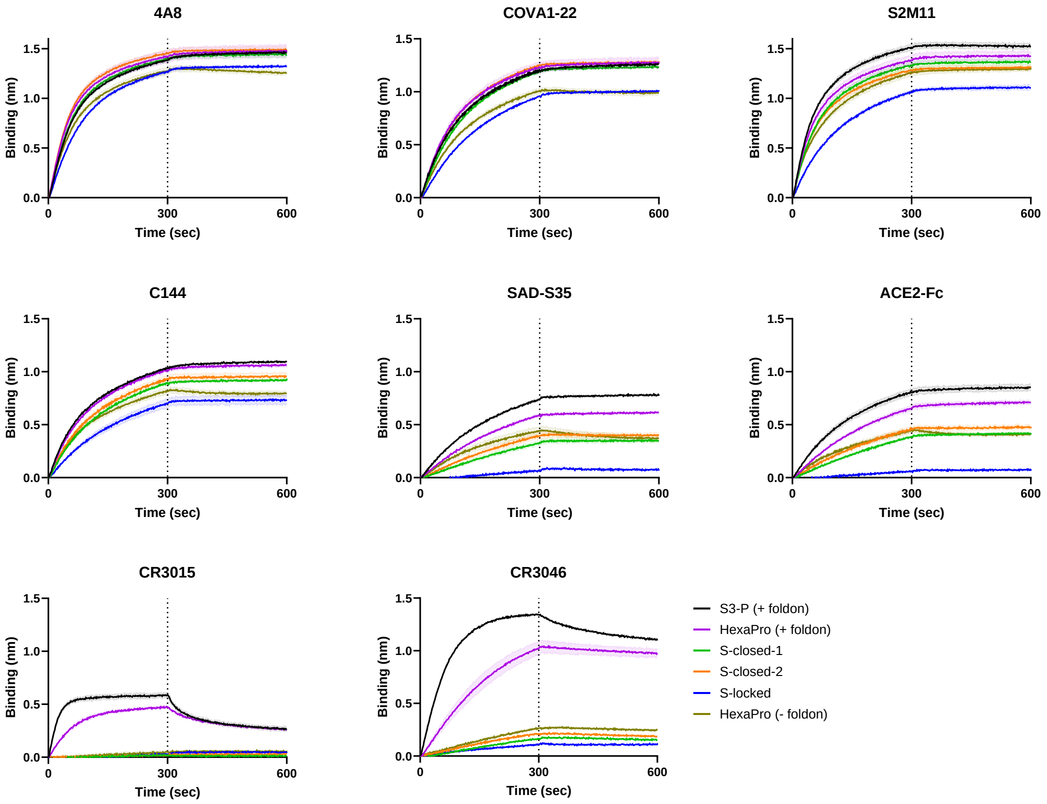


**Supplementary Figure S5. Biolayer interferometry using Octet.** Biolayer interferometry curves of SARS-CoV-2 S proteins binding to ACE2-Fc fusion and diverse antibodies, as indicated above for each panel. The association curves (left of the vertical dotted line) were used to calculate the initial slope $V_0$ plotted in Figures 2D, 2E, 2F and S3D. Right of the vertical dotted line dissociation is measured. Lines depict average of n=3 technical replicates ± standard deviation (shaded area).

**Supplementary Table S1.** SEC-MALS analysis of purified spikes

| Protein | Theoretical Mw[a] (kDa) for trimer without glycans | T=0 | | T=8 weeks at 37°C | | T=8 weeks at 4°C | | | |
|---|---|---|---|---|---|---|---|---|---|
| | | Trimer | | Trimer | | Trimer | | Monomer | |
| | | Mw[a] (kDa) | Rh[b] (nm) | Mw[a] (kDa) | Rh[b] (nm) | Mw[a] (kDa) | Rh[b] (nm) | Mw[a] (kDa) | Rh[b] (nm) |
| S-3P | 409.5 | 466 | 11.00 | 464.2 | 9.8 | 494.9 | 10.5 | NA[c] | NA[c] |
| HexaPro | 409.5 | 453.3 | 9.5 | 462.5 | 9.1 | 454.6 | 9.7 | NA[c] | NA[c] |
| S-closed-1 | 397.0 | 455.6 | 9.1 | 466.5 | 8.8 | 447.7 | 8.8 | 175.3 | ND[d] |
| S-closed-2 | 396.8 | 439.5 | 8.9 | 454.3 | 8.7 | 451.1 | 8.8 | 159.3 | 6.2 |
| S-locked | 397.2 | 462.4 | 9.2 | 456.3 | 8.8 | 471.4 | 8.7 | NA[c] | NA[c] |

[a] Mw stands for molecular weight

[b] Rh stands for hydrodynamic radius

[c] NA stands for not applicable

[d] ND stands for not determined
